# The efficacy and safety of Kuntai capsule combined with leuprorelin acetate in the treatment of endometriosis: A protocol for systematic review and meta-analysis

**DOI:** 10.1097/MD.0000000000025080

**Published:** 2021-03-19

**Authors:** Shuzhen Zhang, Lei Wang, Jing Zhang, Wei An, Li Jia

**Affiliations:** a Hengshui People's Hospital, Hengshui, Hebei Province; b First Hospital of Zibo City, Zibo, Shandong Province, China.

**Keywords:** endometriosis, Kuntai capsule, leuprorelin acetate, protocol, systematic review

## Abstract

**Background:**

Endometriosis (EMS) is one of the common diseases in women, which seriously affects the quality of life of women. Leuprorelin acetate can control the development of EMS, but long-term use can cause perimenopausal symptoms in women. Clinical studies have shown that Kuntai capsule combined with leuprorelin acetate is effective in the treatment of EMS, which can relieve perimenopausal symptoms, but it lacks of evidence-based medical evidence. Therefore, this study aims to systematically evaluate the efficacy and safety of Kuntai capsule combined with leuprorelin acetate in the treatment of EMS.

**Methods:**

CNKI, VIP, Wanfang, Chinese Biomedical Literature Database, PubMed, The Cochrance Library, Embase, Web of Science, and other databases were searched by computer to collect randomized controlled trials of Kuntai capsule combined with leuprorelin acetate in the treatment of EMS. The retrieval time was from the establishment of the database to February 2021. Two researchers screened the literatures and extracted the data and meta-analysis was performed using RevMan 5.3 software.

**Results:**

This study evaluated the efficacy and safety of Kuntai capsule combined with leuprorelin acetate in the treatment of EMS by clinical effective rate, serum sex hormone levels estradiol, follicle-stimulating hormone, luteinizing hormone, visual analogue scale, Kupperman score and incidence of adverse reactions.

**Conclusion:**

This study will provide reliable evidence-based evidence for the clinical application of Kuntai capsule combined with leuprorelin acetate in the treatment of EMS.

**Ethics and dissemination:**

Private information from individuals will not be published. This systematic review also does not involve endangering participant rights. Ethical approval will not be required. The results may be published in a peer-reviewed journal or disseminated at relevant conferences.

**OSF Registration number:**

DOI 10.17605/OSF.IO/AZU47

## 1 Introduction

Endometriosis (EMS) is a common benign gynecological disease in clinic, which is caused by the presence of active endometrial cells outside the uterus.^[1–2]^ In recent years, the incidence of EMS has increased year by year. Ten percent to 15% of women of childbearing age have EMS, and the incidence of EMS in infertile women is as high as 50%.^[3–4]^ The common clinical symptoms of EMS include chronic pelvic pain, dysmenorrhea, and infertility,^[5–6]^ which seriously affect the quality of life of women. Patients can be treated by surgery or drugs. Gonadotropin-releasing hormone agonist (GnRH-a) drugs are known to be used in the clinical treatment of EMS,^[7]^ the development of lesions can be controlled by maintaining the low hormone level in patients, and the condition of patients can be improved.^[8]^ Leuprorelin acetate is a GnRH-a drug, but long-term use will cause perimenopausal symptoms such as insomnia, hot flashes, memory loss, and so on.^[9]^

Kuntai capsule is a commonly used Chinese patent medicine in gynecology, which can be used for diseases such as premature ovarian failure,^[10]^ polycystic ovary syndrome,^[11]^ perimenopausal syndrome,^[12]^ and EMS,^[13]^ etc. Kuntai capsule has the effects of nourishing yin and clearing heat, tranquilizing mind and removing boredom,^[14]^ and can improve perimenopausal symptoms caused by various reasons.^[15]^ Studies^[16–18]^ have found that Kuntai capsule combined with Western medicine in the treatment of EMS to some extent improve clinical efficacy, improve serum hormone levels and alleviate perimenopausal symptoms. Although many clinical trials have shown that Kuntai capsules combined with leuprorelin acetate is effective in the treatment of EMS, there are differences in the efficacy evaluation of various studies, and the results are uneven, which affects the promotion of this treatment to a certain extent. Therefore, this study aims to objectively explore the efficacy and safety of Kuntai capsule combined with leproteroline acetate in the treatment of EMS through systematic evaluation, and to provide a reliable reference basis for the clinical application of Kuntai capsule combined with leproteroline acetate in EMS.

## 2 Methods

### 2.1 Protocol register

This protocol of systematic review and meta-analysis has been drafted under the guidance of the preferred reporting items for systematic reviews and meta-analyses. It will be registered in the open science framework (OSF) (registration number: DOI 10.17605/OSF.IO/AZU47).

### 2.2 Ethics

Since this is a protocol with no patient recruitment and personal information collection, the approval of the ethics committee is not required.

### 2.3 Eligibility criteria

#### 2.3.1 Types of studies

We collected randomized controlled trials of Kuntai capsule combined with leuprorelin acetate in the treatment of EMS, regardless of blinding, publication status, region, but language would be restricted to Chinese and English.

#### 2.3.2 Study objects

Patients with definite diagnosis of EMS,^[19]^ in which nationality, race, age, gender, course of disease, location of onset were unlimited.

#### 2.3.3 Study interventions

The control group was treated with leuprorelin acetate alone, and the experimental group was treated with Kuntai capsule combined with leuprorelin acetate, both for more than 3 months.

#### 2.3.4 Outcome index

1. Primary outcome: the overall effective rate;
2. Secondary outcomes: ① estradiol; ②follicle-stimulating hormone; ③luteinizing hormone; ④visual analogue scale; ⑤Kupperman score; ⑥incidence of adverse reactions.

### 2.4 Exclusion criteria

1. Republished literatures;
2. Review, theoretical research, experimental research, and other non-clinical experiments;
3. Studies in which the control group was treated with leuprorelin acetate and other treatments;
4. Studies published as abstracts and unable to obtain data after contacting authors;
5. Studies in which the data are obviously wrong.

### 2.5 Search strategy

“Zi gong nei mo yi wei zheng” (Endometriosis), “liang bing rui lin”(Leuprorelin), “ kun tai jiao nang” (Kuntai capsule) were searched as Chinese search terms in the CNKI, VIP, Wanfang, Chinese Biomedical Literature Database database; “endometrioma,” “leuprorelin,” “Kuntai Capsules” were searched as English search terms in the PubMed, Embase, Web of Science, the Cochrane Library. The retrieval time was from establishment of database to February 2021. All domestic and foreign literatures on the treatment of EMS were collected. Taking PubMed as an example, the retrieval strategy is shown in Table 1.

**Table 1:** Retrieval strategy of PubMed.

Number	Search terms
#1	Endometriosis[MeSH]
#2	Endometrioses[Title/Abstract]
#3	Endometrioma[Title/Abstract]
#4	Leuprolide[MeSH]
#5	Leuprorelin[Title/Abstract] OR Enantone[Title/Abstract] OR Leuprolide Acetate[Title/Abstract] OR Acetate, Leuprolide[Title/Abstract] OR·Leuprolide Monoacetate[Title/Abstract] OR·Monoacetate, Leuprolide [Title/Abstract] OR Leuprolide, (L-Leu)-Isomer[Title/Abstract] OR Lupron[Title/Abstract]
#6	Kuntai capsule[Title/Abstract] OR KTC[Title/Abstract]
#7	#1 OR #2 OR #3
#8	#4 OR #5
#9	#6 AND #7 AND #8

### 2.6 Data screening and extraction

**Figure 1:**
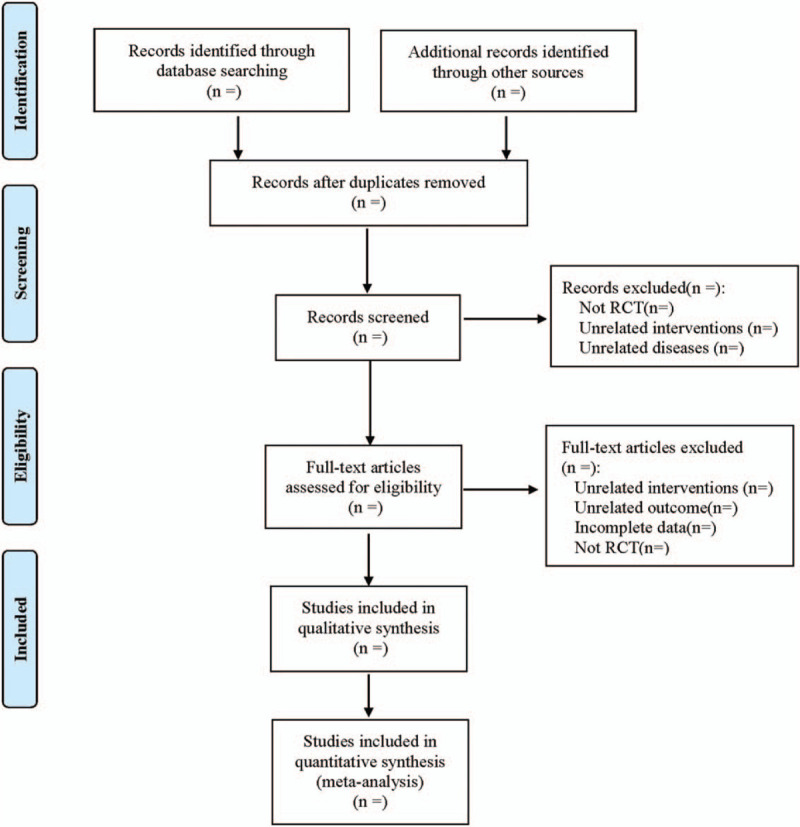
Flow diagram.

Two researchers screened the included literatures by referring to the research selection method of Cochrane Collaboration Network System Evaluator Manual Version 5.0. The 2 researchers independently used EndNote X7 literature management software to screen the literatures. If differences were encountered, they would be discussed and resolved, and if necessary, they would be determined by the third party. Excel 2013 was used to extract the relevant information in the literatures, including: ① basic information (first author, publication time, country, etc.); ② baseline characteristics of the subjects (age, course of disease, sample size, etc.); ③ treatment methods and course of treatment of the control group and the experimental group; ④ outcome index; ⑤ key elements of bias risk assessment. The literature screening process is shown in Figure 1.

### 2.7 Evaluation of literature quality

Two researchers used the Cochrane collaboration's tool for assessing risk of bias to assess the risk bias of the included study, and cross-checked after completion, if there was any disagreement, negotiate with the third party researcher.

### 2.8 Statistical analysis

#### 2.8.1 Data analysis and processing

Meta-analysis was performed using RevMan5.3 software. Relative ratio was used to represent the binary variables; for continuous outcomes, the weighted mean difference was used as the effect size, both expressed with 95% confidence interval. If the heterogeneity among studies was low (*P *≥* *.1, *I*^2^* *≤* *50%), a fixed-effect model was used for meta-analysis. If the inter-study heterogeneity was high (*P < *.1, *I*^2^* > *50%), the source of heterogeneity was further analyzed. If the clinical heterogeneity and methodological heterogeneity were not significant, statistical heterogeneity was considered and the random effects model was used for analysis. If clinical heterogeneity was too obvious and subgroup analysis was not possible, only descriptive analysis would be performed instead of meta-analysis.

#### 2.8.2 Dealing with missing data

Obtain the complete data in the article and contact the author to supplement relevant information. If the author cannot be contacted or the author has lost relevant data, descriptive analysis would be carried out instead of meta-analysis.

#### 2.8.3 Subgroup analysis

Subgroup analysis was carried out according to the age of the patients; subgroup analysis was carried out according to the different stages of the disease; subgroup analysis was carried out according to the different course of treatment.

#### 2.8.4 Sensitivity analysis

In order to judge the stability of the outcome index, sensitivity analysis was used to analyze each outcome index.

#### 2.8.5 Assessment of reporting biases

The funnel plot can be used to evaluate the publication bias. In addition, Egger and Begg test were used for the evaluation of potential publication bias.

#### 2.8.6 Evidence quality evaluation

The Grading of Recommendations Assessment, Development, and Evaluation will be used to assess the quality of evidence. It contains 5 domains (bias risk, consistency, directness, precision, and publication bias). And the quality of evidence will be rated as high, moderate, low, and very low.

## 3 Discussion

EMS is an estrogen-dependent disease,^[20–21]^ and long-term use of leuprorelin acetate can cause perimenopausal symptoms and reduce the quality of life of patients.^[22]^ Therefore, the guidelines suggest that the use of GnRH-a drugs in the treatment of EMS can be combined with Kuntai capsule to reduce perimenopausal symptoms.^[23]^ Kuntai capsule combined with leuprorelin acetate can reduce insomnia, tidal fever and other symptoms, alleviate perimenopausal symptoms, and improve the quality of life of patients.^[24]^

EMS belongs to the category of “tong jing” (dysmenorrhea) and “zheng jia” (abdominal mass) in traditional Chinese medicine. It is caused by qi stagnation, qi deficiency, cold coagulation, liver and kidney deficiency and so on, and the treatment should be mainly tonifying qi and nourishing yin, activating blood circulation, and removing blood stasis.^[25]^ Kuntai capsule is composed of 6 medicines, including Shudihuang (Radix Rehmanniae preparata), Ejiao (Corii Asini), Huangqin (Radix Scutellariae), Huanglian (Rhizoma Coptidis), Baishao (Raidix Paeoniae Alba) and Fuling (Poria), among which Shudihuang (Radix Rehmanniae preparata) can nourish yin and blood, and replenish essence; Huangqin (Radix Scutellariae) and Huanglian (Rhizoma Coptidis) can clear heat and purge fire, remove annoyance and calm the mind; Ejiao (Corii Asini) and Baishao (Raidix Paeoniae Alba) can tonify blood and nourish Yin, soften liver and relieve pain; Fuling (Poria) can induce diuresis for removing edema, nourish heart, and calm mind. Kuntai capsule can inhibit the growth of ectopic endometrial tissue and reduce ectopic endometrial volume by regulating the level of TNF-α and other signaling pathways.^[26]^ Kuntai capsule also has the effects of anti-oxidation and anti-apoptosis, which can repair the ovarian structure and improve the ovarian function.^[27]^ The study found that Kuntai capsule can improve some low estrogen symptoms, such as hot flashes, insomnia, and so on, the clinical effect is remarkable.^[28]^

Through this study, we can systematically evaluate the efficacy and safety of Kuntai Capsule combined with leuprorelin acetate in the treatment of EMS, and obtain the difference in efficacy between Kuntai capsule combined with leuprorelin acetate and leuprorelin acetate alone. We can also learn the adverse reactions of Kuntai capsule in clinical practice, which is conducive to the promotion of clinical use. But this study also has some limitations, because most of the studies are carried out in China, there may be publication bias. This study has only searched Chinese and English literatures, may ignore studies or reports in other languages.

## Author contributions

**Data curation:** Shuzhen Zhang, Lei Wang.

**Funding acquisition:** Li Jia.

**Resources:** Lei Wang, Jing Zhang.

**Software:** Wei An, Li Jia.

**Supervision:** Jing Zhang.

**Writing – original draft:** Shuzhen Zhang, Lei Wang.

**Writing – review & editing:** Shuzhen Zhang, Li Jia.
